# Industry 4.0 and Digitalisation in Healthcare

**DOI:** 10.3390/ma15062140

**Published:** 2022-03-14

**Authors:** Vladimir V. Popov, Elena V. Kudryavtseva, Nirmal Kumar Katiyar, Andrei Shishkin, Stepan I. Stepanov, Saurav Goel

**Affiliations:** 1 Department of Materials Science and Engineering, Tel Aviv University, Ramat Aviv, Tel Aviv 6997801, Israel; 2 Higher School of Engineering, Ural Federal University, 620002 Ekaterinburg, Russia; s.i.stepanov@urfu.ru; 3 Obstetrics and Gynecology Department, Ural State Medical University, 620000 Ekaterinburg, Russia; ekud2019@gmail.com; 4 School of Engineering, London South Bank University, 103 Borough Road, London SE1 0AA, UK; kumarn@lsbu.ac.uk (N.K.K.); goels@lsbu.ac.uk (S.G.); 5 Rudolfs Cimdins Riga Biomaterials Innovations and Development Centre of RTU, Institute of General Chemical Engineering, Faculty of Materials Science and Applied Chemistry, Riga Technical University, 1007 Riga, Latvia; powder.al.b@gmail.com; 6 Department of Mechanical Engineering, University of Petroleum and Energy Studies, Dehradun 248007, India

**Keywords:** Industry 4.0, healthcare, digitalisation, Internet of Things, big data

## Abstract

Industry 4.0 in healthcare involves use of a wide range of modern technologies including digitisation, artificial intelligence, user response data (ergonomics), human psychology, the Internet of Things, machine learning, big data mining, and augmented reality to name a few. The healthcare industry is undergoing a paradigm shift thanks to Industry 4.0, which provides better user comfort through proactive intervention in early detection and treatment of various diseases. The sector is now ready to make its next move towards Industry 5.0, but certain aspects that motivated this review paper need further consideration. As a fruitful outcome of this review, we surveyed modern trends in this arena of research and summarised the intricacies of new features to guide and prepare the sector for an Industry 5.0-ready healthcare system.

## 1. Introduction

The Fourth Industrial Revolution, otherwise known as Industry 4.0, is advancing healthcare to unprecedented comfort levels on the foundation of digitisation, artificial intelligence, and 5G telecommunication [1,2]. In this context, Table 1 summarise various definitions used currently in the context of Industry 4.0 to explain many of its subsystems. These factors have helped in many ways to combat the ongoing crisis the world is facing in the wake of the COVID-19 pandemic [3,4,5,6].

**Figure 1:**
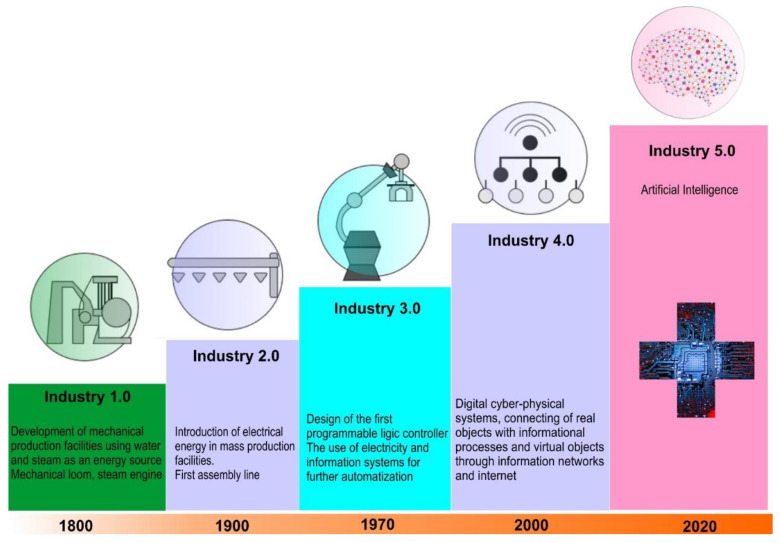
Evolution of industrial developments over time.

**Figure 2:**
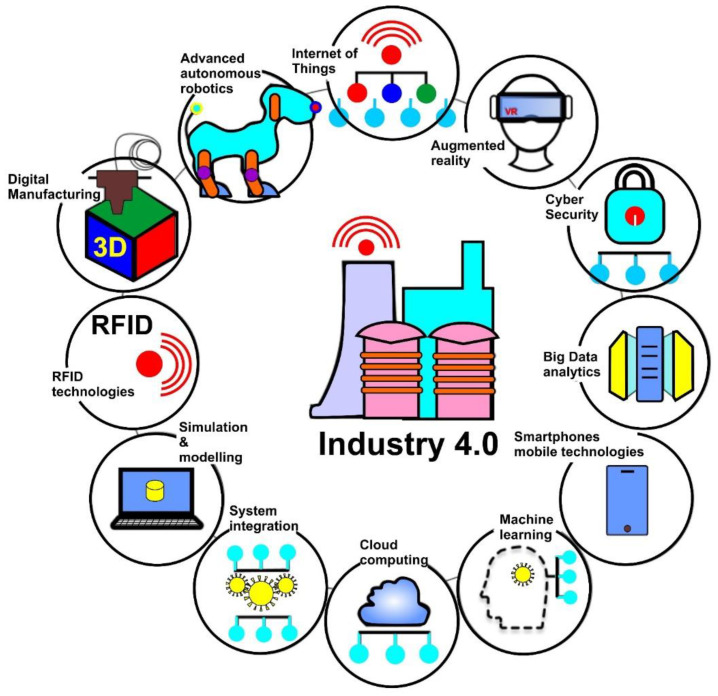
Building blocks of an Industry 4.0 system.

Different digital projects have been developed globally by incorporating digital diagnostic systems which have significantly improved agility in X-ray and MRI investigations. This has, in turn, allowed quick diagnosis of patients’ healthcare data retrospectively as well as clinical anamnesis to provide prompt feedback [7,8]. A question worthy to be asked at this stage is: what is next? The answer to this question primarily drove this review. As shown in Figure 1, the review begins by providing an insight into the interoperable development of the current ecosystem involving people, industry, business, and the government, which forms the backbone of Industry 4.0 in sharp contrast to the previous industrial revolutions. In modern times, machines have become sufficiently intelligent to make decisions in real time and to feed those decisions through cloud-based technologies [9] using neural networks [10,11] and decision-support systems [12]. Figure 2 shows the core components and essential elements of an Industry 4.0 system.

**Table 1:** Definitions of critical elements in an Industry 4.0 system.

Name	Alternative Term	Definition	Ref.
Internet of Things	Industrial Internet of Things; IoT; IIoT	A single device or a system of devices having network access and communication with information networks and the internet.	[13,14,15]
Artificial intelligence	AI; Deep Learning; Machine Learning	AI is a collective term for computer systems that can perceive their environment, think, learn, and can take action in response to stimuli or pre-assigned goals.	[16,17,18,19,20,21]
			[22,23,24,25]
Neural networks	Artificial Neural Network; ANN	A mathematical model or computing system, as well as its software or hardware implementation, built on the principle of organization and functioning of biological neural networks—networks of neurons of a living organism.	[10]
Blockchain	Cryptographic ledger	A continuous chain of blocks containing all the records of transactions and safe distribution among participants.	[26,27,28]
Additive manufacturing	Digital manufacturing; 3D Printing	The process of manufacturing parts, which is based on the creation of a three-dimensional physical object from a digital geometric model, by adding material in a layer-by-layer manner.	[29,30,31,32,33,34]
Advanced materials	Composites; High Entropy Alloys; Hybrid materials	New groups of materials which are out of standard classification—metals/alloys, ceramics, polymers.	[32,35,36,37,38,39,40,41,42,43,44]
Radio-frequency identification	RFID	A communication system that stands for radio frequency identification method. This is a method whose task is to recognize living or inanimate objects using radio waves. Fingerprints, retinas, voice, or clothing are used as Auto-ID.	[45,46]
Big data analytics	Big data; BDA	This technology deals with a large array of data, enabling the derivation information relevant for rapid decision making	[47,48,49]
Digital medicine	Digitalisation in medicine; Hospital 4.0 H-IoT	The collective term for Industry 4.0 technologies used in medicine.	[15,50,51]
Virtual & augmented reality (including medical application)	VR & AR	Perceived mixed reality created with the help of a computer using “augmented” (visual/audio) elements of perceived reality, when real objects are projected in the field of perception.	[52,53,54,55]
Virtual and VR Experiments	VE & VRE	Virtual experiments and experiments with body part surrogates.	[56,57,58]

In fact, the use of deep neural networks has enabled AI to make unprecedented improvements to quality of learning. For example, working with Alexa, Google Search, and Yandex Disc has helped learning over time and the more these tools are used, the more the system becomes trained.

There are numerous examples of use of Internet-of-Things (IoT)-enabled systems which can be seen in day-to-day life. An Amazon store without cash registers or cashiers with the capability to charge users simply based on their body movements is an excellent example, while another involving the use of IoT include Uber, Ola, and GetTaxi. Recently, Lv et al. [59] investigated the issue of quality service and network loading for next generation IoT systems. Additionally, environmental aspects of Industry 4.0 are now also being explored [2,60].

Table 2 highlights state-of-the-art use of advanced technologies in healthcare and medicine revealed by different research papers. This review paper is the first to highlight the prospects of the Industrial Internet of Things (IIoT) in the healthcare sector. Table 2 highlights the novelty of this review paper vis-a-vis increasing interest of the scientific community in this area.

Recently, Austin et al. [7] investigated collaboration between academia, SMEs and digital health industries for the promotion of innovative digital solutions in healthcare. Qadri et al. [15] presented an extensive review of IoT applications in healthcare with careful articulation of the previous literature in this field. They introduced the term H-IoT (Healthcare IoT) to emphasize the importance of IoT in the field of healthcare and medicine. Marques et al. [62] presented a review on IoT applications in healthcare highlighting the need of medical professionals, students, and engineers. They discussed the advantages of IoT platforms in achieving personalized healthcare and developing smart devices for diagnosis and monitoring. They also pointed out the limitations on social readiness [62]. Hau et al. [69] showed how the digital tools of Industry 4.0 could be used to combat COVID-19 pandemic. Von Eiff et al. [51], in their short review, discussed prospects of digitalisation in healthcare. Their work partly discussed digital development and the use of Industry 4.0 tools in medicine development. 

From this brief discussion, the importance of Industry 4.0 in the healthcare sector is obvious. Thus, this review highlights state-of-the-art digitalisation of medicine and healthcare and alludes to the sharp transition this sector is facing while moving towards Industry 5.0. This review also aims to discuss the trends in digital medicine and healthcare and to provide future directions in this area.

## 2. Ingredients of an Industry 4.0 Healthcare System

### 2.1. Internet of Things (IoT)

IoT is a term that refers to any device with network access [13,26,70,71]. Modern devices/objects/networks of objects/systems are equipped with sensors, software, and network equipment. The network equipment and these sensors are capable of compiling and processing data arrays using internet [2,70,72,73,74,75] protocols.

5G has made an enormous impact on IoT technology and economy due to its superior level of connectivity and improved functionality. The key 5G technology drivers are superfast broadband, ultra-reliable low latency communication, massive machine-type communications, high reliability/availability, and efficient energy usage [73,76,77,78].

**Figure 3:**
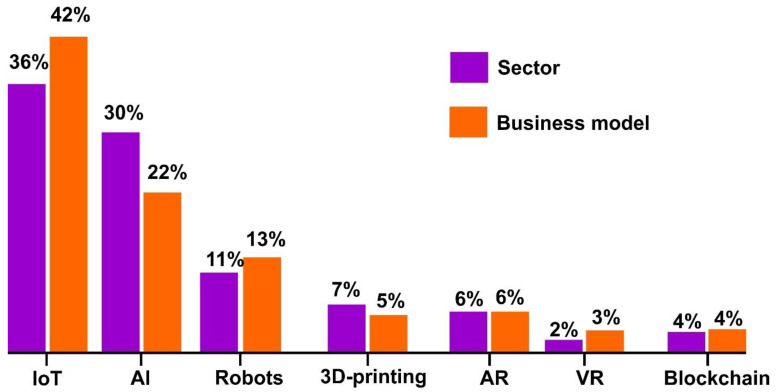
Leading position of IoT in Industry 4.0 structure [80].

The main area of applications of 5G-enabled IoT are the tracking of goods and materials, asset monitoring, remote data collection, self-service systems, remote service delivery systems, real-time market data, and flexible pricing models [71,79]. As per the review of Likens et al. [80], it would appear as shown in Figure 3 that the Internet of Things will lead to be the most promising techniques that will change the gamut for industries and academia in the post-Fourth Industrial Revolution era. 

Most modern industries utilise modelling and simulations for process monitoring, control, diagnosis, optimisation, and design. Industry 4.0 and massive digitisation have made it possible to collect and process large arrays of data, resulting in the development of data-driven decisions and modelling tools [81]. It is worth mentioning that data-driven, statistical, or empirical models do not require broad initial knowledge about the studied system, but strongly rely on the presence of data collected from the process [82]. Modern simulation tools are used for predicting natural disasters which might lead to many victims (e.g., tsunami) [83,84,85,86]. A new trigger for modelling advancement re-emerged in recent years due to the development of machine learning techniques and a variety of Industry 4.0 technologies. Big data and modern modelling and analytical tools provide new horizons even to address old legacy issues and open new scenarios for realising innovative ideas.

### 2.2. Artificial Intelligence

Artificial intelligence (AI) allows computers to learn from their own experience, adapt to given parameters, and perform tasks that were previously only possible for humans. In most AI implementations such as computer chess players or self-driving cars, the role of deep learning and natural language processing is critical. AI allows automation of repetitive learning and searching processes using data acquisition to identify trends. Forms of AI in use today include digital assistants, chatbots, deep learning, and machine learning [16,17,18,19,20,21,22,23,87,88,89].

### 2.3. Big Data Analytics (BDA)

Big data analytics (BDA) is one of the key components of Industry 4.0. Big data technology deals with large arrays of data, enabling the derivation of information relevant for rapid decision-making. The derived data is transformed into the relevant goal-oriented knowledge to help achieve agility in problem solving [47,48]. The successful application of BDA in online trade can be seen through AliExpress, Amazon, and eBay. Technologies for image data are also rapidly developing enabling target recognition, photo filtering, and stereoscopic three-dimensional (3D) contents [90,91,92,93].

### 2.4. Digital Manufacturing and Advanced Materials Processing

One of the main outcomes of the advances in digital manufacturing is 3D printing technology, also called additive manufacturing (AM) [29,30,31,32,33]. AM enables the processing of polymers, ceramics, glass, and metallic alloys. Using approaches such as the Design for Additive Manufacturing (DfAM) [94] and Materials Design by Additive Manufacturing (MaDe-by-AM) [95], novel materials can now be manufactured with ease, which includes tailored composition as well as structural and functionally graded materials [32,35,36,37,38,39,96,97,98]. By shape and composition complexity, the design of new porous materials and metamaterials can also be fabricated. Moreover, the flexibility of maneuvering the printing head allows on-site printing of freeform shapes, which are potentially useful to develop custom-sized implants or prostheses [31,99,100]. Digitalisation of industrial manufacturing is developing due to the implementation of design strategies for new materials development [95,96,101,102]. Now, these additive technologies allow the printing of concrete buildings/structures [103]. Additive manufacturing of concrete structures is much more promising for fast construction in complex natural environments compared to other techniques [104,105,106].

### 2.5. Green Aspects of Industry 4.0

Among other aspects, the environmental aspects of Industry 4.0 deserve a special mention. Some of those aspects in relation to food-water-energy nexus are highlighted below:

1. The survival of humanity will largely depend on how we address the following concerns in the upcoming years:
  - Global energy shortage and depletion of raw materials (energy crisis) [107,108,109];
  - Reduction of arable land, decrease in soil fertility, and food shortage (food crisis) [110];
  - Depleting availability of clean water [111]
  - Catastrophic state of the environment (ecological crisis) [60,112,113,114].
2. Main spheres of life such as industry, transport, the fuel and energy complex, the economy, public administration, and security have taken new forms. This is due to the penetration of digital technologies into everyday life and the development of alternative energy and electrical vehicles [115].
3. Modern industrial development cannot proceed without efficient re-use and recycling procedures [116,117,118,119].

## 3. Digitalisation in Medicine

**Figure 4:**
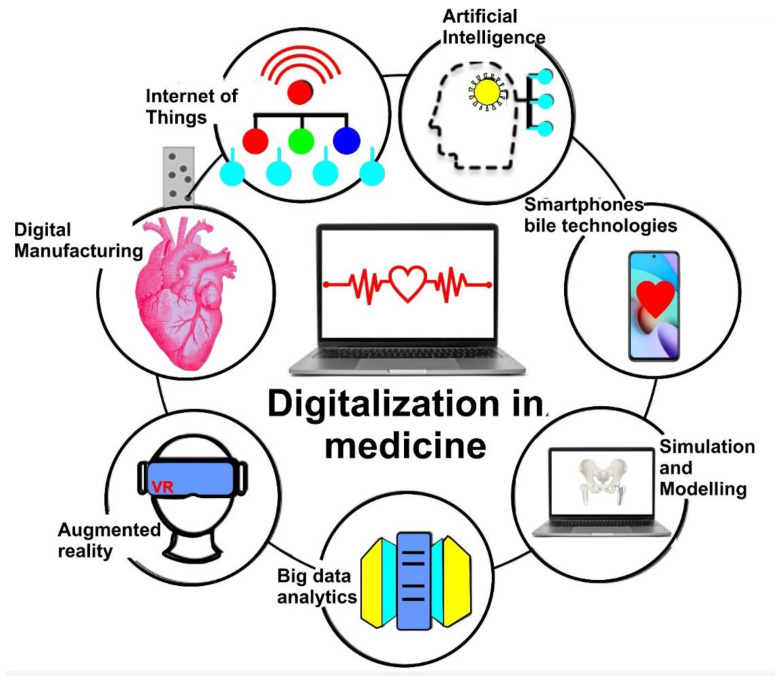
Digitalisation in medicine—main technologies.

The term Medicine 4.0 is closely related to Industry 4.0; it describes the fourth stage in the development of medicine. Modern medicines which emerged around 150 years ago are undergoing a digital journey with the help of robotics, internet and artificial intelligence. The introduction of AI systems in medicine is one of the most important modern trends in world healthcare. Modern medical treatments cannot achieve their full potential without using advanced computing technologies. AI technologies are fundamentally changing the global healthcare system, allowing a radical redesign of the system of medical diagnostics, the development of new drugs, advanced analysis, testing, and treatment to enable advances in the field of transplantation surgeries [50,51,120,121]. Computational simulation using finite element analysis (FEA) is a crucial part of the digitalisation process in medicine [122,123]. FEA allows medical engineers/industrial designers to study many inter-related concepts including, for instance, device stability and durability (e.g., predicting end-of-life of patient-specific implants). FEA enables modelling of stresses within a material under different thermodynamic conditions [124]. In an FEA model, the part is simulated and analyzed using representative physical behavior [122,125,126,127]. Such an approach demonstrates weak areas of the part, and it allows enhancement of the design. Digitalisation and AI generally improve the quality of healthcare services while reducing costs for medical clinics. Figure 4 highlights key technologies enabling digitalisation of medicine.

### 3.1. On-Demand Healthcare

According to Fox et al. [128], consumers are increasingly using online platforms to obtain medical information due to the following reasons:

- 47% wish to know more about their doctor.
- 38% would like to check a hospital and its medical facilities.
- 77% would prefer online medical appointments.

In the new regime of digital economy, medical professionals, just like freelance professionals, can provide their skills, talents, and expertise directly to the patients.

Several healthcare companies provide an online marketplace that connects medical workers directly with the required medical facilities. This results in a much more effective way to provide on-demand medical procedures and services to consumers. In turn, healthcare workers have now become a part of the digital healthcare industry providing patient-oriented treatments [129].

### 3.2. Telemedicine

Telemedicine is a rather modern trend that became especially popular during the COVID-19 pandemic [130]. Such an approach enables support and care of patients in a non-crucial state. Telemedicine minimise the number of contacts between ill patients. Moreover, such educational support is important for chronic patients, and to prevent diseases [131]. According to the data of John Hopkins, before the first global lockdown in March 2020, the number of televisits was approximately 50–70 per month. By May 2020, this number radically increased to 94,000. Moreover, after healthcare services were broadly reopened, the number of monthly televisits remained about 35,000 [132].

Technologically, this kind of telecommunication provided the direct transmission of medical information in various formats (medical history, laboratory data, X-ray images, CT scan results, video images, ultrasound, etc.), as well as real-time video conferencing between medical institutions or a doctor and patients.

The use of telemedicine enabled the provision of consultative medical services in those areas where patients do not have the opportunity to receive the help of focused specialists directly at a medical institution. Telemedicine is of no less importance even in developed countries. With its incorporation, treatment costs have significantly reduced, the quality of diagnostics has improved, and remote monitoring of health has become accessible. This is especially important for elderly patients and patients with chronic diseases. For example, St. Luke University Health Network in Pennsylvania regularly hosts video conferencing to help elderly patients. They recognize that this social group is less likely to use mobile applications and is more comfortable with technologies that target desktops or laptops.

According to the Global Telemedicine Market Outlook, the global telemedicine market reached USD 56.2 billion in 2020 and is expected to reach USD 175.5 billion by 2026. The annual growth rate is about 19.2% [133]. Patient telemonitoring accounts for the main share of 32–48% of the world market (see Table 3). The leading countries in terms of spending on telemedicine and the development of the telemedicine technology market are China and the United States [133].

The global telemedicine market can be segmented according to several criteria, including:

- The nature of remote interaction (clinic–clinic, clinic–patient’s home);
- Technological parameters of interaction (monitoring systems, communication and communication channels, measuring instruments and sensors, video conferencing systems, databases, mobile and “wearable” technologies);
- Purpose of application (medical education, diagnostics, monitoring, consultation, treatment).

Depending on these, different approaches to the design and development of software solutions are augmented and, accordingly, different tools are used. However, as these segments are closely intertwined, the developer must have skills and expertise in a wide variety of development areas, including experience with embedded solutions, mobile cloud technologies, and protocols specific to the medical industry. It can be concluded that for telemedicine technologies to flourish, it is crucial to provide:

- Remote interaction of medical workers with each other, with patients, and/or their legal representatives;
- Identification and authentication of specified persons;
- Documenting their actions during consultation and remote medical monitoring of patient’s health.

### 3.3. Data Privacy & Cybersecurity in Medicine and Healthcare

With progress in big data and its advancement into medical innovation, there are potential risks to patient data privacy [134]. Healthcare is a prime target for cyberattacks, and even with continued investment in cyber security, critical vulnerabilities remain in many of the medical devices that hospitals rely on to treat patients [135]. Modern healthcare requires advanced solutions that reduce risks due to cyberattacks. Alongside this, GDPR patient sensitive data also needs to be protected so that the privacy of patients is not compromised.

For healthcare organisations, it is extremely important to ensure proper handling of patient data not only according to GDPR, but also because it is crucial for transparency with patients [136]. It can be said that the narrow scope of data privacy laws can be an issue. That can be traced to the U.S. health data privacy law (HIPAA), which regulates data between healthcare professionals and patients, but not un-identified data. For example, the data shared with a fitness trainer, tracking from smartphones, and data from various apps can be considered unprotected.

However, the relevant laws have become more and more effective in medical data privacy. The European Union’s General Data Protection Regulation, which passed in 2016, is a good example. EU law now requires data processors and controllers to provide users with their own data, clearly disclose data collection, set high-privacy defaults, and more.

Healthcare gadgets are not fully unprotected, similar to other IoT devices, and that affects data privacy and even the safety of the device. Risks and threats always exist. The trends in the rapid growth of the audience facing cyberattacks can be explained by the growing usage of electronic medical records and an increase in the amount of medical equipment and IoT devices connected to hospital networks. Additionally, the spread of viruses that interfere with the work of not only computers, but also medical devices is a continual problem. However, to eliminate such issues, cybersecurity is actively regulated by healthcare- and government-associated organizations such as the FDA [137]. This results in reduced risk of potential cybersecurity threats in legally marketed healthcare devices. It can be concluded that there is a necessity of regulated procedures to protect patient data [138].

### 3.4. Big Data Analytics (BDA) in Healthcare

BDA in healthcare enables improved diagnostic practice efficiency. Moreover, even therapeutic treatment based on BDA is much more accurate. It is especially relevant for cases involving tumours, including hard cancer pathologies. The main points of extreme importance in this regard are timely diagnosis and accurate choice of treatment [50,121,139].

BDA is advantageous for genetic analysis to compute genetic pathology and generate possible problem-oriented knowledge [121,140]. It should be considered that even superfast computers may take hours to do an intense data analysis which can now be accelerated using GPU computing [141].

BDA helps to decrease the rate of medication errors and to predict future admission rates [50]. It can be concluded that BDA helps to make healthcare more predictive. Predictive analysis about burden on healthcare systems and admission loading allows healthcare providers to make services more effective and to optimize resources for active deployment.

It should also be considered that predictive analysis could help companies to smoothly mobilise manpower resources by predicting possible outbreaks of colds/flu that could lead to manpower shortages.

Pharmaceutical manufacturers are looking to gain access to patient health data and are striking deals with technical companies knowledgeable in BDA, a tool that creates new possibilities for understanding how drugs work in real life. One recent example is the Roche deal announced in 2018, wherein the company acquired all shares of Flatiron Health, a clinical data collection company for cancer patients, by paying USD 2 billion [142]. Examining real-world evidence allows pharmaceutical manufacturers to prove the usefulness and value of their drugs. The most active research in this area is carried out in the fields of oncology, heart disease, and respiratory disorders. Actual drug use data is collected outside of traditional randomized clinical trials, which are today’s gold standard for drug evaluation. Neural networks are now effectively used for the development of automated drug discovery. Researchers and medicinal chemists work together to identify problematic issues and create more proficient models for newer drug design [143]. Advances in interdisciplinary fields that combine computational and genomic technologies are expected to lead to newer horizons in personalized medicine [121,140,144,145].

In the United States, the Human Microbiome Project was launched simultaneously with the renowned Human Genome Project [146]. During its implementation, a special Data Analysis and Coordination Center was created within the framework of the US National Institutes of Health. A joint Chinese–European project MetaHit is being implemented, where active research is being carried out in this direction.

### 3.5. Augmented Reality and Virtual Reality (AR & VR)

Smart glasses with AR functionality allow warehouse workers to achieve a higher level of accuracy. For critical life applications like aircraft production, AR helps manufacturers to precisely assemble and repair planes and to achieve improved reliability during repairs [147]. VR is transforming healthcare and changing the way patients are being treated. For millions of people suffering from chronic pain, VR is a working alternative to drug medication. VR is a safe and efficient treatment for pain and a powerful rehabilitation instrument for anxiety, post-traumatic syndrome, stress, strokes [54]. Healthcare professionals and medical students use VR simulations for improving their skills and for complex surgery planning. The global virtual and augmented reality healthcare market is expected to reach USD 5.1 billion by 2025 [148]. Recently, Nevada Spine Clinic surgeons performed a posterior lumbar fusion procedure using a Medtronic Mazor X robotic platform and an xvision augmented reality headset [149]. This is usually a rather invasive and time-consuming operation, lasting six to seven hours, but in this case, using the xvision headset in tandem with the Mazor X robotic platform, the surgery took less than two hours. The xvision headset allows the surgeon and their team to locate implants more accurately, which would have otherwise taken longer time. Before the procedure, the orthopedic surgeon and neurological surgeon used a robotic platform to carefully plan the exact placement of the implant and screw system. During surgery, an augmented reality headset allows the surgeon to refer to a 3D anatomical plan that has been previously created. As a result, the entire process becomes minimally invasive and much more efficient. The incidence of complications and the recovery time of patients reduced sharply due to the reduction in the time spent in the operation theatre, as did the minor damage to soft tissues compared to that with the open access.

In late September 2021, Kinomatic launched a virtual reality-based scaffolding platform that allows surgeons to develop customized plans for knee and hip arthroplasty [150]. This platform can work with any implant system and can be adapted for a variety of surgical techniques and surgical approaches. The company’s patented VR app allows surgeons to view and manipulate surgical plans in unparalleled detail. The platform supports preoperative modeling for knee and hip arthroplasty using preferred implants, surgical techniques, and specifications. Patients receive high-resolution computed tomography, which is then converted into an accurate 3D model of the patient’s joint after completion of the simulation. The surgeon can determine the exact size and orientation of the implant which will most accurately recreate the natural anatomy of the joint.

VR headsets are used for sports and fitness promotion, and these help children with autism to learn how to orient in the real world [151]. Application of VR training for patients with autism results in improvement of daily living skills [151]. VR and AR possess the potential to help older adults to overcome mobility issues and cognitive ability and socialisation limitations [124]. It was recently shown how the translational potential of VR can be used to reduce suicide risk [152]. VR factors like dissociation and derealisation allowed the simulation of the experience of a suicide opportunity and to reduce this risk. Virtual reality not only helps humans, but also enables investigation of the behavior and environment of animals [55].

### 3.6. VR Experiments

Development in computational technologies have led to new ways of supporting research and development work, which are now also regarded as “virtual experiments”. They are quickly emerging and rapidly developing tools within different applications of virtual reality (VR), including medicine [153]. One can briefly define “VR experiment” (VRE) as an advanced tool of computer modelling. Due to the advantages of modern technology in computation and visualisation, VRE has already started to play a significant role in cases where real experiments are extremely dangerous or prohibitively costly. One such case is related to research in safety, medicine and healthcare. In that aspect, VR experiments can be regarded as non-intrusive and non-invasive. This method is based on the development and utilisation of complex, advanced models of the human body and its parts. These models are further used for testing various “what if” scenarios in both static and dynamic cases, making predictions about how the human body will react to different situations, and how various equipment designed to prevent injuries or help with surgeries will work. Today, such models have become so sophisticated that one can speak about “digital twins” [154,155], and the results of modelling are coming close to experimentation. One of the examples of true success stories in the development of advanced digital twins and virtual experimentation in medical and safety research is modern achievement in the study of traumatic brain injuries and in the related development of modern protection helmets [156,157,158,159,160].

**Figure 5:**
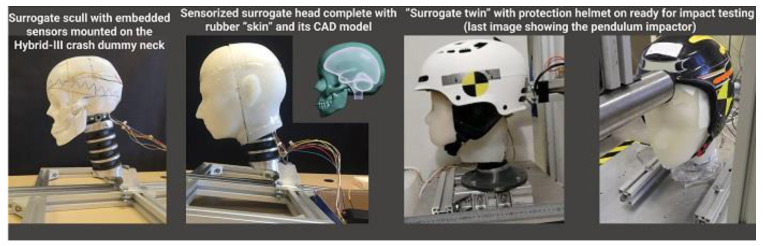
Example of body part surrogate for VRE. Photo courtesy of Prof. A. Koptyug, SportsTech Research Center, Mid Sweden University, Sweden.

Unfortunately, computer modelling in general and virtual experimentation in particular are not free of problems. One of these problems is that a model cannot be tested from within a model. Further developments in this area are represented by “surrogate twins”. These are body part surrogates (physical models) manufactured using additive manufacturing, another modern technology enabled by digitisation [57,58,161]. Such surrogates can have the same shapes and outlines as their digital counterparts, as they use the same CAD files (see Figure 5). Properties of materials used in the surrogate mimic soft human tissues and bones and can be exactly characterized. Surrogates often have encapsulated sensors and experiments in realistic conditions are performed with them. Experiments with surrogate twins are also performed without endangering humans, and collected data are equally useful for both research and development and for cross-validation of both digital and physical models. Significant progress in the application of surrogate twins is achieved in the area of safety research and studies of injury mechanisms [57,58,160,161].

These methods harness the power of digitisation, from scanning shapes to making surrogates of differing complexity with embedded sensors using additive manufacturing and synthetic materials mimicking different tissues. Such an innovative approach allows the design of new devices with a high degree of efficacy. The application of surrogate twins is helpful in both civil and military areas.

### 3.7. Wearable Medical Devices

Wearable medical devices are a modern trend in healthcare. They help to collect health data to monitor patients’ health. These devices provide day-to-day health data and are active monitoring tools compared to once-a-year or once-a-month clinical checkups. 

Medical companies are investing in wearable smart devices that can provide up-to-date monitoring of high-risk patients, preventing major health episodes. According to a recent report, the wearable medical device market is expected to reach USD 46.6 million by 2025, a spectacular jump from approximately USD 8 million in 2017 [162]. Some of the most common of devices include:

- Smart watches with heart rate sensors and exercise tracking.
- Sweat meters used by diabetics to monitor blood sugar levels.
- Smart patches to measure hydration levels, body temperature, heart rate, and other biometric parameters.
- Oximeters measure the amount of oxygen in the blood, which is relevant to patients with respiratory illnesses, e.g., asthma.
- Headphones to monitor blood pressure.
- Biosensors in modern devices are able to not only read pulses and measure steps and calories, but also measure hydration, electrolyte levels, blood pressure; obtain electrocardiogram (ECG) results; and determine muscle load, strength, and fatigue level.

There are many tangible benefits for healthcare companies in spreading the use of wearable devices:

- These devices make patients themselves responsible for monitoring their actions leading to a certain health state.
- People are incentivized to engage in more sports and remain fit by actively monitoring their results, thus setting new goals. Such practices decrease the risk of obesity and reduce the burden on the healthcare system.
- Relevant medical information is on demand for insurance companies to assess the real risks to patients’ health.
- Patients who use technologies for preventive treatment and monitoring are offered discounts on their health insurance.

### 3.8. 3D Imaging and Prototyping 

The whole complex of assisted three-dimensional techniques is used in 3D prototyping as described below:

- 3D visualisation/medical image processing of a medical problem using medical computed tomography, MRI, and X-ray examination tools [120,139]. Machine learning can be used for medical image processing. After the required features from a particular medical case are extracted, this data can be processed for accurate decisionmaking [99].
- 3D-modelling using modern digital tools like Magics by Materialise and analogues. A complex model of a damaged area could be realized for further surgical planning. This is especially relevant for complex cancer cases where a surgeon needs to attempt resection from multiple sides of a tumor.
- 3D-planning digital systems enable online communication between the surgeon and the engineer responsible for 3D printing of the implant.
- 3D-prototyping using polymer printers improves the accuracy of custom-designed metal implants [26,100,146].
- 3D-printing of organs and tissues, also called bio-printing, is a rapidly developing application for meeting the demands of modern transplantology [163,164].

3D printing is used for additive manufacturing of patient-specific metal/ceramics implants; for stereolithography of drug delivery systems; for polymer/metal-based individual prosthesis; and for individual surgeons’ tool designs [121].

### 3.9. Machine Learning & Deep Learning

In biomedicine, machine learning and deep learning are used to simulate human knowledge and for complex analysis of special medical data and biomedical and biophysical processes in the human body [15]. Here, AI works for the systematisation of assisted behavioral processes using complex machine learning (see Table 4).

The main goal of AI in biomedicine is to establish relationships between patients’ health, diagnostics, selected treatment program, and follow-up outcomes. Due to AI, the effectiveness of diagnostics and selected therapeutic treatment could be radically improved [25]. Machine learning for such diagnostic tools as electrocardiography (ECG) and electroencephalography (EEG) supports doctors in screening and recognition of the disease, and possible risks reduction [165]. The use of machine learning enables the processing of the extracted data, such as heart rate, pulse, intervals, variability from ECG/EEG data, etc. A combination of these parameters can help to identify the existence of heart-related diseases [166].

The diagnosis of cancerous tumours by applying AI (deep learning, image classification, object recognition) to MRI images is not inferior to the conclusions of highly qualified radiologists in terms of accuracy. AI algorithms are also an effective solution for proper patient-oriented and patient-specific drug selection.

The implementation of novel digital tools is sometimes referred to collectively as the concept of “hospital 4.0”, emphasizing the digital character of progress in medicine [50,51,121]. Areas of using AI in medicine can be specified as follows:

- At the design level: predicting diseases, identifying groups of patients with high risk of disease, organizing preventive measures.
- At the production level: automation and optimisation of processes in hospitals, automation and improvement of diagnostic accuracy.
- At the promotion level: price management, risk reduction for patients.
- At the level of service delivery: adaptation of therapy and the composition of drugs for each individual patient, the use of virtual assistants for planning a patient’s route in a polyclinic or hospital.

However, at present, the healthcare community has no unanimous opinion of whether digitalisation in medicine is necessary and helpful for all healthcare industries, or if it is being forced on the medical community because of general trends. This means that deep academic research on this topic is in demand by health care specialists [51]. The limitations of digitisation stem from massive hardware requirements. Traditionally, mechanical parts such as the bolts and nuts of an instrument can simply be taken off and replaced, but with electronics this is not the case. Hardware now very quickly becomes obsolete, and software or firmware updates are no longer available after their intended design period. Moreover, the electronic circuits themselves become dated, as they can no longer function at the same speeds as newer hardware. Consequently, despite having the so-called “right to repair”, the repair of digital hardware is almost impossible and is not cost-effective even if pursued. This grim situation is leading the accelerated generation of electronic waste and these aspects require serious consideration in view of the growing focus on a “net zero” economy. 

## 4. Summary

In the post-4th Industrial Revolution era, the term “5th Industrial Revolution” started to be used in scientific reported data [167]. In healthcare, the terms “hospital 4.0” and “medicine 4.0” are gaining popularity by highlighting a new era in medicine- and healthcare-assisted spheres. The main trends in technological and economic development that can be observed are that the advent of the Industrial Internet of Things is a very specific economic and technological evolution that requires new action and will enable incredible development. Now, business strategy and administration need to be customer-oriented through new relationships provided by IIoT. It is also noteworthy that artificial intelligence, big data analytics, and robotics will enable the very fibre of everyday life, especially in safety-critical applications. Additionally, “virtual experiments” and experiments with body part surrogates applied for medicine and healthcare represent the true power of digitisation, from scanning shapes to making surrogates of different complexity with embedded sensors using additive manufacturing and synthetic materials to mimic different tissues. With the use of finite element modeling, “virtual experiments” can achieve newer horizons hitherto unrealised. 

In the time to come, newer forms of prescription will become the most exciting medical advances. Tablets with support for microscopic sensors can provide doctors with the best information about the condition of a patient internal organs. In this regard, digital techniques including 3D printing and digital health devices (IoT) will pave the way for patient-specific, need-oriented, and predictive/provision-based approaches for all spectra of the medical industry. BDA will lead various areas of medical digitalisation, having a significant influence on the healthcare industry’s evolution and development. According to analysts’ forecasts, the market volume of the Internet of Things in medicine will exceed USD 158 billion by 2022. The average market growth rate (CAGR) in the period from 2016 to 2022 was estimated by Market Research Engine experts to be 30.8% [168]. This review identifies the breakthrough technologies of digitalised healthcare as important ingredients.

In the area of big data and modern modelling, the medical industry would benefit from digital tools tailoring existing technology to the needs of the industry, healthcare professionals, and patients.

Among the factors contributing to the increase in medical IoT costs on a global scale are the growing number of chronic diseases, the introduction of favourable initiatives by governments in various countries and the evolution of artificial intelligence technologies. Current assessments suggest that further integration of digital instruments and technologies will improve the efficiency of the healthcare system, the development of patient-oriented innovations, the transformation of business models, and new workspace organization.

It is necessary to note that the digital technologies discussed in this paper relate to the most successful industrial application up to now. However, both international cooperation and organizational efforts are required for deeper research and further digitalisation. Moreover, it is important to mention that higher awareness from society on digitalisation and Industry 4.0 technologies through improved education and development of “digital” professionals will be of great importance.

Future research will cover the aforementioned technologies related to digitalisation in medicine and healthcare, including virtual and VR experiments, biological additive manufacturing, development of cybersecurity, and pandemic predictive big data analysis.

## Figures and Tables

**Table 2:** The key technologies discussed in the recent review publications (adapted and expanded from [15]). Here, “V” stands for the presence, and “X” for the absence of discussion on the relevant topics that make up an Industrial 4.0 system.

Ref.	IoT	IIoT	AI			Blockchain	Digital Manufacturing	VR & AR	Hospital 4.0 H-IoT	RFID	Big Data
			Deep Learning	Machine Learning	Neural Networks						
[15]	V	X	V	V	V	V	X	V	V	V	V
[61]	V	X	X	V	X	X	X	X	X	X	X
[62]	V	X	X	X	X	X	X	X	X	X	X
[63]	V	X	V	V	X	X	X	X	X	X	V
[25]	V	X	V	V	X	X	X	X	X	X	X
[51]	V	X	V	V	X	X	V	X	V	X	V
[64]	V	X	X	V	V	X	X	X	X	X	V
[65]	V	X	X	X	X	X	X	X	V	V	V
[66]	V	X	X	X	X	X	X	X	X	X	X
[67]	V	X	V	V	V	X	X	X	V	X	X
[68]	V	X	X	X	X	X	X	X	X	X	X
This work	V	V	V	V	V	X	V	V	V	X	V

**Table 3:** Telemedicine directions.

Telemedicine Directions	Application
Teleconcilium	Communication between consultant doctors from different medical institutions, or different professional areas, and the attending doctor
Telemonitoring	Monitoring patients with chronic diseases
Teleconsultation	Remote doctor–patient consultations
Medical archive, patient’s personal account	Maintaining and storing patient health records
Data integration	The ability to merge and exchange information between different clinics, health authorities, insurance companies, etc.
Maintaining a register, making an appointment with a doctor	Remote appointment with a doctor
Remote access to equipment	Control over the condition of equipment, remote diagnosis of the patient
Tele-teaching	Conducting lectures, video seminars, conferences, including operating rooms

**Table 4:** Areas leveraging AI in medicine.

Goals	Effect
Analysis (including cross-sectional) of population data, registration data, Omix data, social networks	New correlations for further scientific research and medical applications
Analysis of medical images, creation of a system with an automatic initial level of description and interpretation of results	Improving the speed and quality of medical decision-making
Smart scripts for patient surveys	-
Clinical decision support system (CDSS), platforms for organizing CDSS as services	-
Operational quality control and intelligent benchmarking of healthcare delivery in an institution	Improving the speed and quality of expert work
Control of long-term consequences of medical care	Changing systems for assessing and analysing the provision of medical care
Systems for increasing adherence of lifestyle of citizens and patients to prescribed treatment	Reducing morbidity and improving the effectiveness of treatment
Modelling the activities of a medical organization	Improving the quality of management, optimizing costs
Wearable and other mobile medical devices for remote monitoring	Online/regular monitoring of health indicators
Smart training medical simulators	Improving the quality of training of medical workers
Medical data visualisation, including smart navigation during surgical interventions	Improving the speed and quality of medical decision-making, medical care

## Data Availability

Not applicable.
